# Broad-Spectrum Anti-Infective Activity of Natural Compounds Pyrrolomycins, Marinopyrroles, and Their Analogs

**DOI:** 10.3390/pathogens15020203

**Published:** 2026-02-11

**Authors:** Brianna N. Davis, Clare F. Euteneuer, Kayleen J. Mijangos, Angelique Vargas, Kailey M. Bruha, Paul H. Davis

**Affiliations:** 1 Department of Biology, University of Nebraska, Omaha, NE 68182, USA; 2 Department of Microbiology and Molecular Biology, Brigham Young University, Provo, UT 84604, USA; 3 College of Medicine, University of Nebraska Medical Center, Omaha, NE 68198, USA

**Keywords:** marinopyrrole A, pyrrolomycins, marine drugs, antimicrobials

## Abstract

Pyrrolomycins and marinopyrroles are natural products originally derived from *Streptomyces* spp. that possess potent anti-infective activity against a variety of organisms, including drug-resistant bacteria and eukaryotic pathogens, especially pertinent amid the search for additional antimicrobial agents. These highly halogenated compounds have been proposed to act as protonophores, an uncommon mechanism of action that likely contributes to their broad-spectrum antibacterial activity. To improve efficacy and overcome limitations to clinical transition, promising derivatives of these natural compounds have been synthesized, introducing structural refinements that enhance pharmacological properties while preserving potent anti-infective activity. Recent discoveries demonstrate the potential of pyrrolomycins and marinopyrroles derivatives to serve as broad-spectrum anti-infective agents with efficacy against drug-resistant bacteria, bacterial biofilms, parasitic infections, and some viruses.

## 1. Introduction

Antimicrobial resistance is an ever-increasing global threat. In 2019, it was associated with 5 million deaths worldwide, and in the United States, it is estimated to cause over 2.8 million infections a year [1,2]. With the number of drug-resistant pathogens increasing and resistance rates rising, effective medical treatment is becoming increasingly challenging to provide as more limited treatment options are available. This also makes the administration of chemotherapy and the performance of surgeries and invasive medical procedures much riskier, as the infectious disease threat is much higher [3].

Microbes themselves are one of the major sources of antimicrobial compounds. *Actinomycetes* species account for the source of about two-thirds of current antibiotics from microbial sources, with 80% coming from the genus *Streptomyces* specifically [4]. *Streptomyces* species are known to produce primary and secondary metabolites with unique natural structures [4]. These compounds, either their natural products or synthetic derivatives, account for over 20 FDA-approved antibiotics, including aminoglycosides (streptomycin, kanamycin, neomycin, etc.), tetracyclines (tetracycline, doxycycline, minocycline, etc.), macrolides (erythromycin, azithromycin, etc.), lincosamides (lincomycin, clindamycin), and other antibacterial classes [5]. Two FDA-approved antifungal drugs, nystatin and amphotericin B, were also isolated from *Streptomyces* spp. Most of these compounds were derived from soil-dwelling species, but marine species metabolites constitute a broad range of unique structures that have also shown antimicrobial activity in preclinical studies [4,6].

The pyrrolomycins and larger, related compounds, marinopyrroles, are two groups of natural pyrrole compounds derived from *Streptomyces* species that share many structural similarities [7,8]. Pyrrolomycins are characterized by the presence of a pyrrole ring, while marinopyrroles have a characteristic bipyrrole group. Through chemical modification of naturally obtained compounds, the creation of synthetic derivatives has allowed these compounds to retain or enhance their antimicrobial efficacy in vitro while reducing cytotoxicity. Cytotoxic compounds will be referred to as compounds with cytotoxic concentrations <50 µM. These natural compounds and their analogs have been shown to have antibacterial, antifungal, and anticancer activity [9,10,11].

With the last comprehensive reviews of these dynamic and compelling compound classes being published about a decade ago [12,13,14], and a recent review on their anticancer activity [11], this review aims to provide an update on further published work of specifically the anti-infective properties of these compounds by exploring an emerging understanding of possible mechanisms of action, analyzing broad spectrum and multi-kingdom activity and efficacy, and highlighting promising derivatives that capitalize on structural activity relationship studies with higher efficacy and less toxicity.

## 2. *Streptomyces* spp.

*Streptomyces* is a genus of filamentous, Gram-positive bacteria within the Actinobacteria phylum. These organisms have large genomes that can encode an extensive number of biosynthetic gene clusters (BGCs), which play an important role in their biosynthetic capacity [4]. BGCs are believed to be responsible for producing diverse metabolites with an array of specialized functions that are not necessary for the species’ core metabolism but have allowed for competitive advantages [4]. Genetic processes, such as gene duplication and adaptive selection, have preserved and diversified these BGCs over time, resulting in the production of many compounds considered secondary metabolites [15,16,17]. The designation of secondary metabolites reflects their non-essential roles: the compounds are not produced for growth and development but rather provide ecological advantages [18].

To outcompete other microbes in resource-limited environments, *Streptomyces* spp. produce molecules that are used for communication, protection, and defense against other microbes [19,20]. The evolutionary success seen by these organisms is therefore reinforced by ecological pressures and the need for survival [21]. Furthermore, marine *Streptomyces* live in a more dynamic, higher stress environment, which is thought to contribute to their production of secondary metabolites with unusual halogenation and functional group patterns, which may enhance or provide novel bioactivity [22,23].

## 3. Pyrrolomycins and Marinopyrroles

Pyrrolomycins (PM) are a family of natural compounds isolated from the fermentation of *Streptomyces* spp. and *Actinosporangium* spp. (originally classified separately but later reclassified under *Streptomyces*) [24,25,26,27,28,29,30]. They are characterized by a singular pyrrole ring and a molecular weight of ~250–420 g/mol.

**Figure 1:**
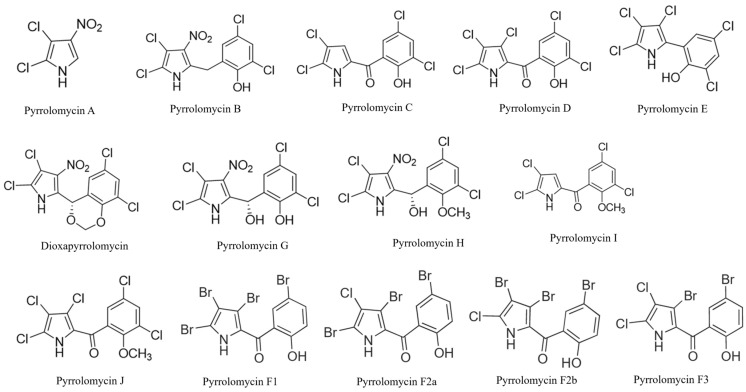
Natural Pyrrolomycins.

The compounds isolated from *Streptomyces* spp. include PM A, B, C, D, E, F, G, H, I, J, and dioxapyrrolomycin (Figure 1). PM A–E were first isolated from *Actinosporangium vitaminophilum* SF-2080, which was later reclassified as a *Streptomyces vitaminophilus* [24,28,30]. PM C has also been isolated from *Streptomyces fumanus* (culture LL-F42248) [31] and PM D from *Streptomyces* spp. [32]. All subclasses of PM Fs, F1, F2a, F2b, and F3, were originally isolated from a novel *Actinosporangium vitaminophilum* species, now *Streptomyces vitaminophilum* [33]. These four natural compounds in the PM F subclass are similar in structure, varying only in the number and arrangement of chlorine and bromine atoms around the pyrrole ring [33] (Figure 1). Dioxapyrrolomycin was isolated from *Streptomyces* sp. MG796-AF7 [34] and *Streptomyces fumanus* LL-F42248 [31], and PM G–J were isolated from *Streptomyces fumanus* LL-F42248 [35]. Recently, PM-like metabolites were identified from *Micromonospora* sp. RB23, but this review will focus on *Streptomyces*-derived PMs and derivatives [36].

Key structural features of PM compounds include their pyrrole core with three modifiable positions and ionizable groups, such as the pyrrole -NH and phenolic -OH (Figure 1) [37]. Five of the PMs (A, B, G, H, and Dioxapyrrolomycin) also possess a nitro (-NO_2_) group at position three of the pyrrole ring. Many of the compounds (C, D, I, J, and all Fs) also contain a salicylic acid group characterized by an aromatic ring connected to the pyrrole by a reaction with the carboxyl group. All PMs are highly halogenated with chlorine and/or bromine. The position and degree of halogenation are strongly associated with their antimicrobial activity as halogenated compounds can form covalent interactions, alter drug binding affinity, cause membrane permeabilization, and range in lipophilicity [38]; however, excessive halogenation may increase cytotoxicity [39].

**Figure 2:**
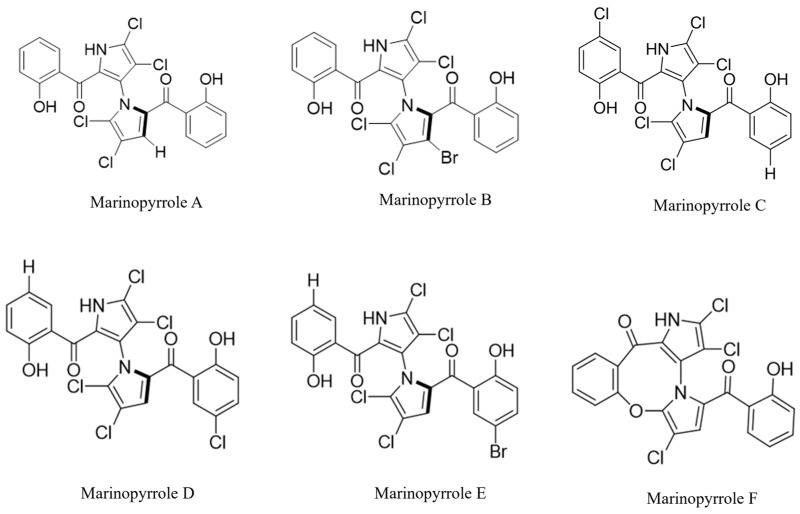
Natural Marinopyrroles.

Marinopyrroles (MAR) are considered structurally related to PMs as they contain a bipyrrole structure. Thus, natural MARs have a greater molecular weight compared to PMs, averaging above ~420 g/mol. Naturally isolated compounds in this family include MAR A, B, C, D, E, and F (Figure 2).

In 2008, MAR A and B were isolated from a novel *Streptomyces* sp. (CNQ-418) found off the coast of California [7]. High-performance liquid chromatography (HPLC) was used to reveal the two related molecules. MAR C, D, E, and F are also isolated of *Streptomyces* sp. CNQ-418 [40]. They share similar structural characteristics to MAR A; however, they possess different levels of halogenation (Figure 2). MAR F differs in that it has an 8-membered ring, connecting one of the salicyloyl groups to its opposite pyrrole group and has less halogenation than the other MARs (Figure 2) [40].

While the complete biosynthetic mechanisms for these compounds are not fully delineated, PMs as secondary metabolites are known to originate from two biosynthetic gene clusters also found in *Streptomyces* (or previously *Actinosporagium*) species. By performing single-crossover disruptions of two specific genes, *dox17* and *dox8*, it was found that PMs were no longer produced, indicating their involvement in PM biosynthesis [41]. In addition, the biosynthetic origin of this nitro group was proposed to result from nitrogen scavenging [42]. Summaries of chemical synthesis have been described previously [14]. Recently, reported synthesis of PM C and the PM F’s has been achieved through microwave-assisted organic synthesis, with yields of 95% and ~66%, respectively [43,44].

MARs form their characteristic bipyrrole structure through a homocoupling reaction catalyzed by MpyBGCs. The homocoupling reaction occurs through flavoproteins, determined to be FADH_2_-dependent halogenases Mpy10 and Mpy11 [45]. Multiple synthetic methods for MAR A have been determined. The full synthesis of MAR B has only been described once [46]. The first total synthesis of MAR A, also considered to be the primary synthetic method [47,48,49,50,51,52,53,54], was achieved in 2010 via a nine-step process with an overall 30% yield [55]. A five-step synthesis was also reported with an overall yield of 16% starting from aminopyrrole [56]. There has been limited investigation into synthetic methods for MAR C–F, as they exhibit less antibacterial and anticancer activity [57].

## 4. Mechanisms of Action

The most groundbreaking findings for natural PMs and MARs in the last 10 years are their proposed mechanisms of action, suggesting targets including bacterial membranes and specific proteins. All of these studies were published post-2016.

Multiple theories as to the potency of PMs against bacteria have been put forth. PMs have been hypothesized to possess protonophore activity, or the facilitation of the transfer of protons across a membrane. In doing so, protonophores disrupt the cell’s proton gradient, which can inhibit nutrient acquisition, motion, and ATP synthesis [58]. When evaluated for mechanism of action, these compounds, especially PM C and D, were found to rapidly depolarize bacterial membranes and could move protons across a planar lipid bilayer current [8]. The phenyl hydroxyl group in these compounds’ structure is believed to play a large role in this activity; based on its chemical properties, it is surmised that it can shuttle protons easily across the cytoplasmic membrane of bacteria. The molecule triclosan exhibits similar characteristics as a protonophore and contains a phenyl hydroxyl group [59].

A closer examination of the protonophoric effects of PMs demonstrated their mitochondrial uncoupling activity, specifically depolarization of the inner mitochondrial membrane and dysregulation of oxidative phosphorylation. Overall, PM C was more active than PM D due to its more efficient penetration of the outer mitochondrial membrane; however, PM D was more potent at depolarizing the mitochondria when the outer mitochondrial membrane was removed [32]. Both compounds were more potent than the well-known protonophore CCCP [32]. PM D is more hydrophobic than PM C because of its chlorine substituent, which may contribute to its ability to perform as a stronger protonophore and disrupt bacterial membranes with more potency (Table 1 and Table 2).

Further studies propose that PMs have multiple mechanisms and cellular targets beyond solely protonophoric activity. Biological activity of PMs may not be only due to protonophore action, but their buildup in the cellular membrane, disrupting overall bacterial function. This lipophilic-driven disruption may interact synergistically with protonophore activity to enhance antimicrobial efficacy [37].

One study showed that in *Staphylococcus*, PM C targeted teichoic acids on cell wall, lipids in cell membrane, post-translation modifications of proteins, and oxidative stress regulation [60]. Wall teichoic acids were more resistant to modifications and hydrolysis. The hydrophobic PMs interact with both polar heads and non-polar lipid tails in the membrane, acting as a bridge for the polar heads and allowing the depolarization of the membrane, while membrane fluidity was found to be severely limited. Protonophoric effects can cause oxidative stress, while PMs with NO_2_ groups can also engage in one-electron reactions to induce ROS. Additionally, oxidative stress relievers like intracellular thiol and staphyloxanthin were rendered unable to handle the stress through nucleophilic aromatic substitutions performed by PMs. Phosphorylation of proteins was also downregulated upon PM C treatment. Susceptible *Staphylococcus* created a thickened and more rigid membrane to counteract the PM effects, enhancing the PM effects, leading to bacterial death [60]. Multi-targeting compounds may lower the risk of antibiotic resistance as overcoming all mechanisms, especially membrane-targeted mechanisms, is more difficult.

As biofilm antagonists, PM C, as well as other synthetic PMs, were found to target and inhibit Sortase A (SrtA), a key enzyme used by many Gram-positive bacteria to anchor surface proteins to their cell wall, which plays an important role in adhesion and biofilm formation. This may allow PMs to interfere with bacterial colonization and pathogenesis rather than having direct bactericidal effects. Further, PM F2a, which has more potent inhibition of biofilm formation than PM C, was found to alter the activity of murein hydrolase, an enzyme that breaks down the bacterial cell wall, allowing for cell growth, biofilm formation, and maintenance. PM F2a diminished the overall activity of murein hydrolase when added to bacterial cultures, leading to inhibited biofilm formation [44]. These two potential targets may explain reduced pathogenesis in treated Gram-positive organisms in biofilm or in vivo models but are likely insufficient to be a cause of reduced IC_50_ concentrations in vitro.

Gram-negative resistance to PMs is frequently observed. The proposed mechanism of resistance is the presence of a TolC-dependent AcrAB-independent efflux pump, which acts to actively remove the compound from the organism. It is believed that Gram-negative bacteria contain target(s) for PMs, but that efflux effects reduce PM concentration, thus preventing bactericidal activity [8].

While PMs and MARs have similar core structures, the physicochemical properties of the bipyrrole may address mechanistic differences. The main proposed mechanism of action for MAR A as an antibacterial, similar to PMs, is as a protonophore. MAR A acts as a protonophore by dysregulating the proton gradient of the cell, which disrupts the membrane polarization. Experimentally, MAR A was found to localize to the bacterial membrane after entry and cause both membrane depolarization and a decrease in intracellular pH within minutes [61]. Instead of pore formation in the membrane, hydrophobic MARs can shuttle protons across the lipid bilayer in opposition to the proton motor force needed for ATP synthesis and cell viability [61]. This mechanism removes the electrochemical gradient, depleting the cell of energy and eventually leading to death. MARs are also highly halogenated and mainly hydrophobic due to the lack of polar groups, making them more lipophilic than PMs. Given this information and their larger size, MARs may exhibit slower diffusion through membranes but stronger and more stable partitioning once embedded [7,61]. MARs may be weaker or have a delayed response compared to PMs, but may be a more efficient protonophore [8,61].

## 5. Antimicrobial Activity of Natural Pyrrolomycins

Natural PMs have exhibited anticancer [11] and anti-infective activity, specifically antibacterial and antifungal. The potency and efficacy of these compounds are discussed in relation to their minimum inhibitory concentrations (MICs) needed to inhibit bacterial or fungal growth, or half-maximal inhibitory concentration to inhibit growth of parasites, viruses, insects, or human cell lines (IC_50_).

From the category of Gram-positive bacteria, natural PMs have been tested against 6 different genera: *Bacillus*, *Corynebacterium*, *Enterococcus*, *Listeria*, *Staphylococcus*, and *Streptococcus* (Table 1). Generally, PM C, D, and F1 had the most activity against Gram-positive bacteria. Many of these values were published before 2016, but this review will emphasize findings from after 2016 (bolded in table) [8,44,60,62].

**Table 1:** Antibacterial Activity of Natural Pyrrolomycins (Gram-positive). Findings since 2016 are bolded.

Notable Condition	Organism (# of Strains Tested)	Compound	Results/MIC/IC_50_ (μM)	Reference
Gram-positive	*Bacillus anthracis* (3)	PM A	8.6	[30]
		PM B	0.3	[30]
		PM C	0.3	[28]
		PM D	≤0.07	[28]
		PM E	≤0.2	[28]
		PM F1	<0.1	[33]
		Dioxapyrrolomycin	2	[34]
	*Bacillus cereus* (1)	Dioxapyrrolomycin	4.1	[34]
	*Bacillus subtilis* (3)	PM D	≤0.003	[63]
		Dioxapyrrolomycin	2.03 (NRRL B-558)–4.07 (PCI 219)	[34]
	*Corynebacterium bovis* (1)	Dioxapyrrolomycin	1	[34]
	*Enterococcus faecalis* (2)	**PM C**	**1** **.5**	[62]
		PM D	0.03	[63]
	*Enterococcus hirae* (1)–previously classified as *Streptococcus faecalis*	PM A	34.5	[30]
		PM B	1.1	[30]
		PM C	1.2	[28]
		PM D	≤0.07	[28]
		PM E	5.1	[28]
		PM F1	<0.1	[33]
		Dioxapyrrolomycin	0.7	[31]
	*Listeria monocytogenes* (1)	PM D	0.03	[63]
	*Micrococcus luteus* (3)	Dioxapyrrolomycin	<0.5 (FDA 16)–4.1 (PCI 1001)	[34]
	*Staphylococcus aureus* (13)	PM A	17.3	[30]
		PM B	0.6	[30]
		**PM C**	**0.3 (2 strains)–19.1 (702)**	[8,28,60,64]
			**IC_50_ biofilm ATCC 25923: 0.92**	[44]
		**PM D**	**≤0.003 (4 strains)–0.07 (2 strains)**	[8,28,60,63,64]
		PM E	5.5	[28]
		PM F1	0.02 (ATCC 25923)–≤0.1 (209 JC-1)	[33,64]
		**PM F2a**	**IC_50_ biofilm ATCC 25923: 0.009**	[44]
		PM G	21.5	[35]
		PM H	2.6	[35]
		**PM I**	**8.9**	[8]
		**PM J**	**2.7–4.0**	[8,35]
		Dioxapyrrolomycin	0.08 (2 strains)–4.1 (4 strains)	[31,34]
	MRSA (2)	PM C	≤0.4	[62]
		PM G	21.5	[35]
		PM H	5.2	[35]
		PM J	5.4	[35]
	*Staphylococcus* *epidermidis* (5)	PM A	34.5	[30]
		PM B	0.3	[30]
		PM C	≤0.08 (ATCC 14990)–38.5 (DSM3269)	[28,64]
		PM D	≤0.003 (3 strains)–0.07 (ATCC 14990)	[28,63,64]
		PM E	5.1	[28,64]
		PM F1	≤0.002 (DSM 3269)–0.004 (RP62A)	[64]
	***Staphylococcus warneri* (1)**	**PM C**	**1.0**	[60]
		**PM D**	**0.06**	[60]
	***Staphylococcus xylosus* (1)**	**PM C**	**>100**	[60]
		**PM D**	**16.6**	[60]
	*Streptococcus agalactiae* (1)	PM D	≤0.003	[63]
	***Streptococcus pneumoniae* (1)**	**PM C**	**0.3**	[8]
		**PM D**	**0.02**	[8]
		**PM I**	**8.8**	[8]
		**PM J**	**1.1**	[8]
	***Streptococcus pyogenes* (1)**	**PM C**	**≤0.4**	[62]

Recent data demonstrated that PM C and PM D exhibited strong activity against *Enterococcus faecalis*, a primary cause of urinary tract infections and wound infections [65], with an MIC of 1.5 μM and 0.03 μM, respectively [28,62]. This is significant because *Enterococcus* spp. has intrinsic resistance to multiple classes of antibiotics including cephalosporins, trimethoprim–sulfamethoxazole, β-lactams, and aminoglycosides [66]. Furthermore, *Streptococcus* spp. is responsible for bacterial pharyngitis, pneumonia, scarlet fever, and more severe complications such as streptococcal toxic shock syndrome [67,68]. In a recent study, it was found that PM C and D are most effective at inhibiting *S. pneumoniae* and *S. pyogenes* with MICs less than 0.4 and 0.02 μM, respectively [8,62]. While pneumococcal conjugate vaccine has significantly reduced disease worldwide, *S. pneumoniae* strains that are not covered by the vaccine are increasing in antibiotic resistance [69], necessitating further work on developing robust antibiotics.

Notably, natural PMs were tested against both drug-sensitive and drug-resistant *Staphylococcus* spp., one of the primary causes of boils and abscesses on the skin and sepsis [70]. All were shown to exhibit moderate to high activities. PMs C, D, and F1 had the lowest MIC values for *S. aureus*, which were between 0.003–0.3 μM [60,64]. PM C and F2a were tested on *S. aureus* biofilms specifically, with PM F2a being a very potent antibiofilm compound with an IC_50_ of 0.009 μM [44]. Immunocompromised patients or medical device recipients are at greatest risk for *S. aureus* biofilm colonization [71]. Many classes of antibiotics are not able to penetrate and treat biofilms, while those that do are facing increasing antibiotic resistance, making PMs like PM F2a a promising compound to consider in further biofilm studies, including in combination treatment approaches [71]. PM C and D were also tested against other clinical isolates of multi-drug resistant (MDR) strains of *Staphylococcus* spp.; PM D was 6–16 times more potent than PM C [60].

Overall, PMs demonstrate greater antibacterial potency and range against Gram-positive bacteria than Gram-negative bacteria. However, a few natural PMs, specifically PM A and F1, have shown moderate to strong activity against Gram-negative bacteria (Table 2). More recent findings (since 2016) support this general observation (Table 2).

**Table 2:** Antibacterial Activity of Natural Pyrrolomycins (Gram-negative). Findings since 2016 are bolded.

Notable Condition	Organism (# of Strains Tested)	Compound	Results/MIC/IC_50_ (μM)	Reference
Gram-negative	*Aeromonas* sp. (1)	Dioxapyrrolomycin	16.3	[34]
	***Acinetobacter baumannii* (1)**	**PM C**	**19.2**	[8]
		**PM D**	**8.3**	[8]
		**PM I**	**>147**	[8]
		**PM J**	**>134**	[8]
	*Aeromonas punctata* (1)	Dioxapyrrolomycin	65	[34]
	*Aeromonas salmonicida* (1)	Dioxapyrrolomycin	16.3	[34]
	*Citrobacter diversus* (1)	Dioxapyrrolomycin	>333	[31]
	*Citrobacter freundii* (1)	PM A	35	[30]
		PM B	35	[30]
		PM C	>308	[28]
		PM D	17.4	[28]
		PM E	>325	[28]
		PM F1	12.4	[33]
	*Enterobacter cloacae* (1)	Dioxapyrrolomycin	>333	[31]
	*Erwinia aroideae* (1)	Dioxapyrrolomycin	>130	[34]
	*Escherichia coli* (11)	PM A	35	[30]
		PM B	35	[30]
		**PM C**	**18.5 (BW25113)**–308 (NIHJ JC-2)	[8,28]
		**PM D**	**8.3 (BW25113)**–16.7 (NIHJ JC-2)	[8,28]
		PM E	>325	[28]
		PM F1	0.1 (2 strains)–12.4 (NIHJ JC-2)	[33]
		PM G	86 (imp)–1490 (WT)	[35]
		PM H	10.4 (imp)–332 (WT)	[35]
		**PM I**	**>148**	[8]
		**PM J**	**>134**	[8,35]
		Dioxapyrrolomycin	16.3 (NIHJ JC-2)–>333 (clinical isolate)	[31,34]
	*Klebsiella pneumoniae* (3)	PM A	69	[30]
		PM B	35	[30]
		**PM C**	**83 (LMG 2095)**–308 (PCI602)	[8,28]
		**PM D**	**13.9 (LMG 2095)**–17.4 (PCI602)	[8,28]
		PM E	>325	[28]
		PM F1	12.4	[33]
		**PM I**	**>147**	[8]
		**PM J**	**>134**	[8]
		Dioxapyrrolomycin	>333	[31,34]
	*Morganella morganii* (1)	Dioxapyrrolomycin	>333	[31]
	*Mycobacterium smegmatis* (1)	Dioxapyrrolomycin	8.1	[34]
	***Mycobacterium tuberculosis* (1)**	**PM C**	**9.2**	[8]
		**PM D**	**8.3**	[8]
		**PM I**	**17.7**	[8]
		**PM J**	**10.7**	[8]
	*Proteus mirabilis* (2)	PM A	35	[30]
		PM B	35	[30]
		PM C	>308	[28]
		PM D	4.3	[28]
		PM E	Not determined	[28]
		Dioxapyrrolomycin	>130	[34]
	*Proteus morganii* (1)	PM A	35	[30]
		PM B	35	[30]
		PM C	>308	[28]
		PM D	17.4	[28]
		PM E	Not determined	[28]
	*Proteus vulgaris* (1)	PM A	35	[30]
		PM B	35	[30]
		PM C	>308	[28]
		PM D	4.3	[28]
		PM E	20	[28]
		PM F1	6.2	[33]
		Dioxapyrrolomycin	>130	[34]
	*Providencia stuartii* (1)	Dioxapyrrolomycin	>333	[31]
	*Providencia rettgeri* (2)	Dioxapyrrolomycin	65 (GN 466)–>130 (GN 311)	[34]
	*Pseudomonas aeruginosa* (4)	PM A	69	[29]
		PM B	35	[29]
		**PM C**	**102 (PAO1)**–>308 (MB-3829)	[8,28]
			**IC_50_ biofilm ATCC 15442: 236**	[44]
		**PM D**	**35 (PAO1)**–70 (MB-3829)	[8,28]
		PM E	>325	[28]
		PM F1	25	[33]
		**PM F2a**	**IC_50_ biofilm ATCC 15442: 79**	[44]
		**PM I**	**>147**	[8]
		**PM J**	**>134**	[8]
		Dioxapyrrolomycin	130 (A3)–333 (clinical isolate)	[31,34]
	*Pseudomonas fluorescens* (1)	Dioxapyrrolomycin	65	[34]
	*Pseudomonas lachrymans* (1)	Dioxapyrrolomycin	>130	[34]
	*Salmonella enteritidis* (1)	Dioxapyrrolomycin	>260	[34]
	*Salmonella typhi* (2)	PM A	17.3	[30]
		PM B	35	[30]
		PM C	>308	[28]
		PM D	17.4	[28]
		PM E	>325	[28]
		PM F1	12.4	[33]
		Dioxapyrrolomycin	>130	[34]
	*Serratia marcescens* (4)	PM A	35	[29]
		PM B	70	[29]
		PM C	>308	[28]
		PM D	35	[28]
		PM E	>325	[28]
		PM F1	6.2	[33]
		Dioxapyrrolomycin	>333	[31,34]
	*Shigella dysenteriae* (1)	Dioxapyrrolomycin	>130	[34]
	*Shigella flexneri* (1)	Dioxapyrrolomycin	>130	[34]
	*Shigella sonnei* (2)	PM A	35	[29]
		PM B	35	[29]
		PM C	>308	[28]
		PM D	35	[28]
		PM E	>325	[28]
		PM F1	12.4	[33]
		Dioxapyrrolomycin	>130	[34]
	*Vibrio anguillarum* (1)	Dioxapyrrolomycin	8.1	[34]
	*Xanthomonas citri* (1)	Dioxapyrrolomycin	260	[34]
	*Xanthomonas oryzae* (1)	Dioxapyrrolomycin	8.1	[32]

While limited testing of natural PMs against Gram-negative bacteria has more recently occurred, PM C, D, I and J were tested against notable Gram-negative pathogens such as *A. baumannii*, *E. coli*, *K. pneumoniae*, and *P. aeruginosa*, the most effective being PM D with MICs of 8.3, 8.3, 13.9, and 35 μM, respectively [8]. *P*. *aeruginosa* is an opportunistic pathogen that is resistant to many antibiotic drugs and also creates resistant biofilms [72]. PM C and F2a were also tested against *P. aeruginosa* biofilms, but were not effective compared to their efficacy on *S. aureus* biofilms [44]. Also newly tested, against *Mycobacterium tuberculosis*, the pathogen which causes tuberculosis [73], PM C, D, I, and J all exhibited moderate activity with MIC values of 9.2, 8.3, 17.7, and 10.7 μM, respectively [8].

Although PMs have been primarily tested as antibacterials, some previous studies have demonstrated their efficacy against other organisms, such as fungi and eukaryotic parasites. Only PM A and D showed mild efficacy against fungi [28,30]. Dioxapyrrolomycin, PM C, and PM D have been tested as mammalian anthelmintics as well; however, only dioxapyrrolomycin exhibited significant activity against *Haemonchus contortus*, clearing 99.9% from lambs at 12.5 mg/kg [74]. Recent studies have not focused on identifying more susceptible fungi or helminths. While PMs do possess moderate to strong efficacy against various pathogens, they have limitations that have prevented them from being investigated further in clinical trials. For example, it has recently been noted that the insensitivity of certain bacteria, such as *M. tuberculosis*, to PM C and D is likely due to the presence of bovine serum albumin (BSA) and/or fetal calf serum (FCS) in growth media [8]. PMs bind strongly to albumin and are therefore strongly serum sensitive; with the removal of albumin, *M. tuberculosis* was 10 times more susceptible to PMs [8]. As albumin is the most abundant protein circulating in human blood, PMs in the bloodstream may bind to albumin instead of performing antibacterial activity [75]. Additionally, when supplementing bacteria media with BSA and/or FCS, the selectivity of PMs for bacteria decreases as their cytotoxicity to human cell lines (HepG2 and HEK293) is similar to antibacterial activity [8]. Consequently, caution must be taken when comparing anti-infective studies that use different serum concentrations since the selectivity indexes may be misrepresented.

Also, a barrier to clinical development, natural PMs are considered moderately to highly toxic in vivo, as most show LD_50_ values < 50 mg/kg, posing a strong limitation for clinical translation [28,30,31].

## 6. Antimicrobial Activity of Pyrrolomycin Derivatives

Like their natural parent compounds, derivatives of PMs show strong antimicrobial activity, especially against Gram-positive bacteria. Moreover, there is evidence of improved activity against Gram-negative bacteria, biofilms, and non-bacterial pathogens compared to their parent compounds while mitigating toxicity (Table 3). Results of PM E and dioxapyrrolomycin derivatives tested on insects and fungi are omitted from the table as they were all reported before 2016 [76,77].

Previous synthetic derivative development focused on optimizing PMs for greater efficacy against specifically MRSA, Gram-negative bacteria, and fungi [25,78,79]. More recent work has focused on combatting drug-resistant pathogens, including MRSA, testing antibiofilm activity, and evaluating anti-infective potential beyond bacteria.

A subset of halogen-substituted derivatives was tested previously [64], but more work has been done to synthesize new analogs, elucidate mechanisms of action, and demonstrate antibiofilm activity. Structure activity relationship studies aim to find the optimal combination and positioning of chlorine, bromine, and nitro groups at available sites (three sites on pyrrole and often two on the phenol, ortho and para to the hydroxyl group). These derivatives most closely resemble PM D and the PM F series.

Compound **5d** carries a nitro group as a substituent on the pyrrole core. Its MIC against *S. aureus* was found to be 0.05 μM with a minimal bactericidal concentration (MBC) of 7.5 μM [37,60]. Against *P. aeruginosa*, **5d** had an MBC of 30 μM [37]. Roughly as potent as its parent compound PM D but more potent than PM C, **5d** underwent further mechanistic evaluation against sensitive and drug-resistant *Staphylococcus* spp. [60], focusing on protonophoric activity, regulation of membrane fluidity, and oxidative stress. The presence of the nitro group was associated with observed increased rigidity of the bacterial membrane and enhanced protonophoric activity [60]. The nitro group functions as a strong electron-withdrawing group and is more acidic, likely making it a stronger uncoupler.

Natural compounds PM D and all the PM F compounds exhibited activity against *Staphylococcus* biofilms [12,44,64]. Derivatives **1d**, **1h**, and **1i**, which carry either bromo- or chloro- substituents at the five sites, were evaluated for antibiofilm activity [44]. Compound **1d** (also known as **1** in [37] and **I** in [64]), with all five sites containing a bromo group, is a potent anti-staphylococcal compound with an MIC of 0.005 μM. Against *S. aureus* biofilm, it is highly effective, with an IC_50_ value of 0.004 μM [44]. Compound **1h** (also **III** in [64]) has four chloro substituents and a single bromo substituent which is on the pyrrole group. Its MIC for *S. aureus* ranges from 0.002–0.05 μM, indicating high potency [64]. As an antibiofilm agent, **1h** is effective in vitro against *S. aureus* with an IC_50_ value of 0.1 μM; however, its IC_50_ value against *P. aeruginosa* biofilm is 170 μM [44]. Compound **1i** also has one bromo substituent; however, its positioning on the fourth site of the pyrrole makes it a more potent antibiofilm agent than **1h**, with IC_50_ values of 0.02 and 18.6 μM for *S. aureus* and *P. aeruginosa*, respectively [44].

Compound **4** (also known as RL002 in [83] and Pyrrolomycin F4 in [62]), is an analog with a fluoro and a bromo group on the pyrrole ring. It has an MIC as low as 0.2 μM for MRSA, compared to 0.4 μM for its parent compound, PM C [80]. Compound **4** was evaluated for biofilm inhibition against *S. aureus*. At 23 μM (twice the MBC), it reduced biofilm formation by 84% after 24 h, 97% after 48 h, and 99.9% after 120 h [80]. Its cytotoxic IC_50_ concentration on HeLa cells exceeded 100 μM, and when administered to mice orally or via intravenous injection, the elimination half-life was approximately 6–7 h, with Compound **4** up to 72 h after administration [80].

Beyond antibacterial activity, Compound **4** has been assessed against the protozoan parasite *Toxoplasma gondii*. Compared to the gold-standard drug pyrimethamine, Compound **4** was more than three times as potent, with an IC_50_ value of 0.17 μM compared to 0.61 μM [83].

Another fluorinated derivative, Compound **6** (also **1** in [84] or **18** in [81]) contains a fluoro group and two bromo groups on the pyrrole ring. It is less potent than Compound **4** as an anti-MRSA compound, with an MIC of 0.4 μM [80]. However, it has been investigated more broadly as an antiviral agent, used in a comparison study between a phenotypic assay and a more standard ATP assay (CellTiter-Glo) for drug discovery for Zika virus [84]. Compound **6** was potent against ZIKV and protected neural stem cells from virus-induced cell death, with an EC_50_ of 8.56 μM [84]. Notably, no cytotoxicity was observed in Vero cells at concentrations up to 100 μM [84].

Thiazolyl-halogenated derivatives were synthesized by inserting a thiazole group between the pyrrole and the phenol moieties. The most well studied is Compound **8c**, which demonstrated an MIC of <0.2 μM for Enterococcus faecalis, Streptococcus pyogenes, and two different Enterococcus faecium VRE strains [62]. However, **8c** showed little effectiveness against MRSA or any other S. aureus resistant strains, with no mechanism proposed to explain this phenomenon [62].

Compound **8c** was also tested in vivo on *S. pyogenes* infections in *Galleria mellonella* moth larvae [62]. As insects, these larvae lack an adaptive immune system; however, their innate immune response is thought to be similar to that of vertebrates. These organisms reproduce quickly, allowing for large-scale studies to be performed, overall serving as a useful model for initial in vivo data collection [85]. As compared to PM C and the no-treatment control, the larvae treated with Compound **8c** displayed higher endpoint survival and a significantly longer median survival time of 36 h rather than 24 h, highlighting the efficacy and low toxicity in vivo within the parameters of this model [62]. The derivative was also evaluated for metabolic bioavailability in various liver microsomes (rat, dog, human, monkey, and mouse) to model how compounds are broken down in the body. **8c** was found to have poor stability in human and dog microsomes, as stability was below 20%, and moderate stability in rat, mouse, and monkey microsomes: 65.70%, 43.30%, and 36.90%, respectively [62]. This recent study demonstrates that simpler models can help predict mammalians in vivo success.

Additional thiazolyl-halogenated derivatives, **9b**, **10a**, and **10b**, effectively disrupted biofilms formed by MRSA strain Mu50, VRE vanB strain, and *Pseudomonas aeruginosa* strain PA01 [62]. Notably, all three compounds disrupted *P. aeruginosa* biofilms despite the MIC values > 470 μM against this organism [62]. It is hypothesized that their lipophilicity, along with high halogenation, contributes to these results, causing increased membrane permeability, as well as alterations in the proton gradient [86].

Similarly, halogenated benzoyl–pyrazole derivatives of PM D, namely **17a**, **17d**, and **17h**, yielded MIC values against *S. aureus* and drug-resistant strains of MRSA and VISA between 0.1–0.2 μM [82]. The derivatives **17d** and **17h** significantly reduce biofilm formation by MRSA strain USA300 after 24 h, as visualized by crystal violet staining at 0.4 μM and 0.2 μM, respectively [82].

## 7. Antimicrobial Activity of Natural Marinopyrroles

Anti-infective activity of marinopyrroles, with a focus on antibacterial activity, has been most pursued with MAR A (Table 4). Other natural MARs (MAR B, C, and F) exhibit some antibacterial activity [40,87]; however, their activity is limited compared to MAR A and will not be discussed in detail in this review [57].

MAR A has been tested against Gram-positive bacteria species from 5 genera: *Bacillus*, *Listeria*, *Streptococcus*, *Staphylococcus*, and *Enterococcus*. While most *Staphylococcus* data is from before 2016, much of the data on the other Gram-positive bacteria and Gram-negative bacteria comes from after 2016.

In terms of Gram-positive bacteria recently assessed for susceptibility to MAR A, *Listeria ivanovii*, a pathogenic species, and *S. pneumoniae*, had MICs of 0.5 and 0.3 μM, respectively [61]. *E. faecalis* and *E. faecium* are the most pathogenic species of *Enterococcus* against which MAR A has been tested, which produced MICs of 8 and 13.3 μM, respectively [61]. Against the non-pathogenic species *E. durans*, the MIC of MAR A was 4 μM [9]. Therefore, *Enterococcus* spp., in general, are less susceptible to MAR A than other Gram-positive bacteria. The limited efficacy of MAR A is consistent with the fact that *Enterococcus* spp. has intrinsic resistance to many antibiotic classes [66,90], highlighting limitations that novel antibiotics have yet to overcome.

For most Gram-negative bacteria, the MICs of MAR A were almost always greater than 64 μM, indicating that they are not susceptible [9,54,61,87]. However, a group of Gram-negative bacteria that possess lipooligosaccharides (LOS) instead of lipopolysaccharides (LPS) on their outer membrane was recently found to be more susceptible than other Gram-negative bacteria, with MIC values from 0.04–11.8 μM [9]. This group includes *Campylobacter jejuni*, *Haemophilus influenzae*, *Moraxella catarrhalis*, *Neisseria gonorrhoeae*, *Neisseria meningitidis*, and *Neisseria mucosa* [9,87]. It is hypothesized that the shorter and less hydrophobic LOS form a weaker barrier than LPS that allows hydrophobic MAR A to penetrate the bacterial membranes of LOS-producing Gram-negative bacteria [9].

Collectively, MAR A is not as effective against a wide range of Gram-negative bacteria, likely due to structural features that make them more resistant to antibiotics, including complex outer membrane and efflux pumps [91,92]. Natural MARs, therefore, are not likely candidates for further investigation against LPS-producing Gram-negative bacteria.

Additionally, MAR A was tested on two species of *Mycobacterium:* pathogenic *M. tuberculosis* and non-pathogenic *M. smegmatis*. They were both resistant to MAR A, with MIC values of 25 and 50–100 μM, respectively [89]. This resistance is possibly due to the intrinsic defenses and regulatory mechanisms that *Mycobacterium* species are known to possess [93,94].

MAR A was evaluated as a potential antiparasitic agent for *Toxoplasma gondii*, the causative agent of toxoplasmosis, which can lead to devastating infections in immunocompromised individuals and developing fetuses [83,95]. Against a Type 1 RH strain, MAR A was able to inhibit the growth of the parasite with an IC_50_ of 0.31 μM, roughly twice as potent as the gold-standard drug pyrimethamine (IC_50_ of 0.61 μM) [83]. Based on these results, further investigation of MAR A was conducted in acute *T. gondii* murine models, where mice with lethal infections showed improved survival after being treated with MAR A at 5, 10, and 20 mg/kg [83]. At 5 and 10 mg/kg, mice exhibited 71.4% survival, while at 20 mg/kg, 85.7% of mice survived [83]. At lower concentrations, mice showed minimal side effects; however, as treatment concentrations increased, the mice became more irritable and lethargic [83].

Even with the efficacy of MAR A as an anti-infective agent in vitro, especially for Gram-positive bacteria, there are two main hurdles to clinical use. In vitro studies have shown that MAR A is rendered ineffective in the presence of ≥20% serum. Against a strain of MRSA, the MIC value for MAR A was over 240 times higher in 20% serum than in 0% serum, 189 μM compared to 0.7 μM [87]. In addition, anti-*Toxoplasma* effects were inhibited when MAR A was tested in the presence of 20% serum [83], suggesting that serum binds strongly to MAR A, likely sequestering and reducing the amount of free compound to act on the pathogen. However, it is worth noting the 70–85% survival experienced by mice treated with MAR A in a *T. gondii* compared to 15% survival in untreated mice [83]. This suggests that serum sensitivity may be overcome or may be compartment specific. Further serum studies need to be conducted to determine the serum effects in the treatments of other pathogens during in vivo experiments.

Secondly, MAR A has shown toxicity to human cell lines, but most of which are cancerous cell lines [7,11,52,88]. However, when tested against a non-cancerous human cell line, HFF (human fibroblast fiber), MAR A had an IC_50_ of >50 μM, which suggests minimal cytotoxicity to non-cancerous human cells [83]. Cytotoxicity assays using more non-cancerous cell lines are essential to verify this finding and confirm low-toxic effects.

## 8. Antimicrobial Activity of Marinopyrrole Derivatives

Derivatives of the parent compound MAR A have been synthesized to maximize efficacy against bacterial pathogens and to explore their potential for broad-spectrum activity further as antibacterial, antiviral, and antiparasitic agents (Table 5). While previously designed for efficacy in *S. aureus*, recent work has focused on determining bacterial targets of the derivatives and adapting derivatives for efficacy on viruses and parasites [48,49,83,96].

MAR A derivatives were designed with *S. aureus* as the main bacterial target. The most well-studied MAR A derivative is a symmetrical derivative defined by trifluoromethyl groups on each end of the benzoyl ring. This compound has received much attention and is labeled differently across publications: compound **33** in [54], compound **41** in [97], MA-D1 in [49], compound **3** in [48], and MPA-CF3 in [96]. It will thus be referred to as MPA-CF3. This derivative has an MIC of 0.2–0.4 μM on MRSA, about twice as potent as its parent compound [54].

The bis-trifluoromethyl groups on this compound are strong electron-withdrawing groups that produce a compound that is more lipophilic with increased acidity and molecular stability, possibly leading to the increased potency observed [54]. Through proteomic analysis, binding assays, and rescue experiments, it was proposed that GlmS is a direct target of MPA-CF3 [49]. GlmS is an enzyme in bacteria that is part of the peptidoglycan production pathway, which is a component of the bacterial cell wall; MPA-CF3 binding is hypothesized to disrupt cell wall synthesis [49]. The MIC for MPA-CF3 against MRSA remained the same even after 14 generations in sub-MIC treatment, suggesting a low propensity for inducing resistance [49]. Furthermore, MPA-CF3 retained potency against linezolid, vancomycin, and teicoplanin-resistant MRSA [49]. It has not been shown if MPA-CF3 also has protonophoric activity similar to MAR A. When administered as a topical agent on mice against a skin infection of MRSA, both 1% and 10% MPA-CF3 reduced bacterial levels to be comparable to pre-infection [49]. When challenged with a systemic MRSA infection, MPA-CF3 at 0.5 and 1 mg/kg led to 80% survival in mice after 7 days, whereas vehicle only had 20% survival [49].

The bis-trifluoromethyl symmetrical MAR A derivative with potent antibacterial effects has also been the focus of the antiviral derivatives. MPA-CF3 was tested against enteroviruses and flaviviruses using human rhabdomyosarcoma (RD) and baby hamster kidney (BHK) cells, respectively [48]. MPA-CF3 was effective against enteroviruses in RD cells with IC_50_ values ranging from 0.01–0.06 μM; however, cytotoxicity concentration at 50% (CC_50_) against host cells was 0.12–0.25 μM, indicating a low therapeutic window where antiviral activity may also lead to cytotoxic effects. MPA-CF3 was not potent against flaviviruses in BHK cells, and no CC_50_ was determined [48]. It should be noted that the host cell line, human RD, is a cancer cell line, while BHK cells are non-cancerous, possibly explaining why a CC_50_ was found in the cancer cell line only. Using a chemoproteomics approach, COPZ1, coatomer subunit zeta-1, was shown to be the likely target of MPA-CF3 in enteroviruses. COPI vesicles with a COPZ1 subunit are used to shuttle virus material from the Golgi back to the ER, a process essential for virus replication. It is suggested that MPA-CF3 inhibits viral replication by binding to and destabilizing COPZ1 [96].

Compounds **15**, **19**, **32**, and **33**, from Xiao et al., are all derivatives of MPA-CF3 [48]. Compound **15** was especially potent against enteroviruses with IC_50_ values ranging from 0.01–0.03 μM. Compound **19** exhibited activity against flaviviruses with IC_50_ values ranging from 0.25 to 0.84 μM. Compounds **32** and **33**, which include aliphatic chains with sulfur atoms, were most potent against ZIKV and DENV with IC_50_ values of 0.07–0.23 μM. Given the results of these synthetic analogs, it was suggested that the presence of sulfur atoms may increase antiviral activity. Conversely, steric hindrance in tetrazole fragments, additional nitrogens, and increased length of the carbon linker between sulfur and nitrogen heterocycles were suggested to contribute less to antiviral activity [48]. Although the COPZ1 mechanism has been proposed for enteroviruses, there is still a need for further mechanistic studies to detail MPA-CF3 effects against other viral families.

Beyond bacteria, MAR A derivatives have been tested against the parasite *Toxoplasma gondii*. RL125 is the asymmetrical monochlorinated derivative that had previously demonstrated potent activity against MRSA (named Compound **1a** in [53]), potentially due to the chloro-substituent at the meta position to the hydroxyl group on only one benzoyl ring, a withdrawing group that may increase solubility and lipophilicity [83]. RL003 is similar to Compound **4** mentioned in PM derivative section earlier but with two bromo substituents instead of the fluoro group. In vitro, both RL125 and RL003 were more than twice as potent against the active stage parasite compared to MAR A (IC_50_: 0.3 μM), with an IC_50_ range of 0.09–0.16 μM [83]. Notably, these compounds were also greater than 3 times more potent than the standard drug, pyrimethamine, whose IC_50_ was 0.61 μM [83,98]. When tested against the cyst-forming dormant form of the parasite, known as bradyzoites, RL003 was able to inhibit growth with an IC_50_ of 0.2 μM [83]. This is a notable finding because current drugs used to treat toxoplasmosis target the active stage, while none can treat the dormant stage [99]. Efficacy against both stages of the parasite life cycle emphasizes the unique abilities of this compound.

These same derivatives, RL003, and RL125, were tested against *T. gondii* in vitro in the presence of 1%, 10%, 20%, 30%, and 50% serum [83]. RL003 was the least affected by the change in serum levels, with an IC_50_ of 0.03 μM in 1% serum and 0.15 μM in 50%, only a 5-fold increase. RL125 was more impaired in presence of serum; however, the IC_50_ at 50% was only 0.38 μM, which was still below that of the gold standard pyrimethamine (0.61 μM) [83]. Limited serum sensitivity was also seen when this compound was tested against MRSA tested earlier [53]. RL003 and RL125 had IC_50_ values greater than 50 μM against human foreskin fibroblast (HFF) cells, suggesting low toxicity to human cells [83].

## 9. Summary of Recent Findings

Novel work since 2016 on PMs, MARs, and their derivatives has revealed their multi-faceted mechanisms of action, chemical modifications with greater potency and decreased toxicity, and new susceptible pathogens.

For both PMs and MARs, the supported primary mechanisms of action are as protonophores, disrupting bacterial membrane potential leading to cell death [8,61]. Secondary mechanisms have been investigated including modifications to the cell wall, steric buildup in the membrane, and induction of oxidative stress [37,60]. The hydrophobic nature of these compounds drives their ability to interrupt the membrane. Compounds that have multiple mechanisms of action are less likely to induce bacterial resistance, as has been shown with derivative MPA-CF3 [49]. PM C and F2a also have defined mechanisms for disrupting Gram-positive biofilms through specific enzymatic targets [44]. It is supposed that other specific molecular targets exist for these compounds and their derivatives, prompting more mechanistic evaluations into specific binding partners.

For natural PMs, Gram-positive bacteria responded best to PM C and to a greater effect to PM D, potentially due to the chlorination at all five substituent locations instead of only four [12]. Higher degrees of chlorination in other compound classes are correlated with increased membrane permeability, and overall halogenation in PMs, MARs and their derivatives increases acidity and hydrophobicity, which can enhance protonophoric activity [8]. In the case of synthetic derivatives, the fluorinated, chlorinated, and brominated-substituted derivatives were able to rival or outperform their parent compound in antibacterial efficacy, especially against MRSA [25,79,80]. Halogenation also seemed to improve biofilm inhibitory properties [62,64,80,82]. Especially in the case of fluorinated analogs, it was proposed that the presence of fluorine atoms acting as electron-withdrawing groups may serve to stabilize the pyrrole, resulting in the observed decrease in toxicity and increased efficacy against bacteria [80]. Indeed, the most characterized synthetic derivative of MAR A is the bis-trifluoromethyl compound with activity against MRSA, other drug-resistant Gram-positive bacteria, and viruses. Moving forward, bis-trifluoromethyl MAR A derivatives could be designed and tested against Gram-positive bacteria and other organisms to increase potency, molecular stability, and in vivo pharmacokinetic properties.

Though PMs have been studied for decades, recent innovative chemical scaffolds can continue to improve on these compounds [62,82]. Thiazole groups (five-membered ring with nitrogen and sulfur atoms) are a common structure in antibiotic compounds but require being used in combination with more potent antibacterial compounds for efficacy [62]. They have also been proposed to reduce drug toxicity effects [100,101]. By putting a thiazole group between the pyrrole and the benzoyl/phenol groups, it was reported that efficacy increased against generally more resistant bacteria (VRE, *S. pyogenes*) [62]. The additional tests on *G. mellonella* larvae demonstrated lowered toxicity in an animal model [62]. Pyrazoles (five-membered ring with two adjacent nitrogen atoms) were also tested in place of the pyrrole structures in PMs. Pyrazoles are found in compounds used clinically as COX inhibitors and have moderate antibacterial effects. By coupling pyrazoles and the benzoyl ring, efficacy was improved against MRSA strains and MRSA biofilms [82]. Future studies could focus on incorporating chemical scaffolds found in current drugs into the basic PM structure to continue increasing efficacy and selectivity for pathogens. In vivo toxicity and pharmacokinetics also need to be more fully considered and improved upon with derivative design.

Structure activity relationship studies on MARs showed that conserving the hydroxyl group on the benzoyl/phenol ring leads to greater efficacy, likely because it is the functional group that can most easily perform protonophoric activity [61]. Previously, many other symmetrical and asymmetrical MAR A analogues were designed; however, few had efficacy greater than that reported for parent compound MAR A except when simply increasing halogenation [56,87,88,97]. Potential avenues of investigation include optimizing the compounds with higher halogenation. Conversely, using highly halogenated chemical structures found in currently approved drugs as templates with the MAR core structure could be explored, similar to recent work with the thiazole and pyrazole groups in PM derivatives. Efforts should be made to lower toxicity instead of solely improving efficacy. Only one recent paper studied decreased serum sensitivity; further studies should investigate the effects of serum on the most potent compounds in vitro and in vivo, as recent results show that in vitro sensitivity may not completely hinder efficacy in vivo [83].

Another novel development is the broader range of pathogens identified with susceptibility to natural and synthetic PMs and MARs. The antiviral potential of these compounds has been demonstrated in flaviviruses and enteroviruses [76,84]. PMs have the properties of BH3 mimetics, mimicking various pro-apoptotic proteins [11,102,103]. Viruses possess the ability to modulate apoptotic proteins in host cells, preventing apoptosis, allowing them to effectively replicate within a host cell—thus, the BH3 mimetic effects of PMs could regulate apoptosis [104]. COPZ1 was identified as a direct target of MPA-CF3 in enteroviruses [96]. Future directions could include testing more viral families and designing derivatives that are specific for inhibiting viruses, as was the MPA-CF3 compound and its analogs.

*T. gondii* is a protozoan parasite with sensitivity to MAR A and derivatives [83]. The protonophoric effect observed in bacteria is potentially causing both membrane and mitochondrial dysfunction in this parasite. Testing MAR A and other potent derivatives (such as MPA-CF3, **5d**, Compound **6**, and **8c**) against other parasites could prove insightful, especially against other protozoans and more specifically apicomplexans such as *Plasmodium* spp. (causes malaria), which are related to *T. gondii* and often are sensitive to anti-Toxoplasma compounds [105].

Though new evidence suggests that some Gram-negative bacteria (LOS-producing) can be susceptible to MAR A, no efforts have been made to synthesize analogs with enhanced activity on Gram-negative bacteria [9]. Previously synthesized derivatives could be adapted for their efficacy first, but perhaps conjugating MAR A or a selected derivative with chemical structures with known Gram-negative effects could lead to more progress. Additionally, cotreatment strategies with LPS inhibitors could be more rigorously investigated to improve the performance of MAR A and analogues on LPS-producing bacteria [106]. Recent work on PMs has not focused on optimizing for anti-Gram-negative activity, though some derivatives were able to inhibit Gram-negative biofilms. Investigating PM and MAR derivatives for broader antibiofilm properties could be promising.

## 10. Conclusions

With the ever-present global threat of infectious diseases in humans, future treatment will rely heavily on the discovery and development of novel anti-infectives. PMs and MARs exhibit promising characteristics demonstrating efficacy against a variety of organisms. The main proposed mechanism of these compounds as protonophores contributes to antimicrobial activity and improves efficacy against drug-resistant bacteria. While limitations, including cytotoxicity and serum sensitivity, have hindered their ability to progress to the clinical stage, notable improvements in the understanding of structure activity relationships have allowed for the creation of potent derivatives with improved results. The unique multi-kingdom broad-spectrum activity of these compounds addresses relevant clinical issues, making them fascinating candidates in antimicrobial drug discovery. Future directions include further investigation of compound mechanisms and scope of activity, SAR understanding specific to each pathogen class, and evaluation of in vivo pharmacodynamic and pharmacokinetic parameters to drive further optimization.

## Figures and Tables

**Table 3:** Antimicrobial Activity of Pyrrolomycin Derivatives. Findings since 2016 are bolded.

Compounds/Series Tested	# of Analogs Tested	Derivative Source	Classification	Organism Tested	Best MIC/IC_50_ Result (μM)	Reference
2′,3′,3′-triiodoallyl-substituted heterocyclic aromatic derivatives	14	PM A	Gram-positive	*Bacillus anthracis* (1)	1.6	[78]
				*Staphylococcus aureus* (1)	12.8	
				*Staphylococcus epidermidis* (1)	11.1	
			Gram-negative	*Citrobacter freundii* (1)	5.9	
				*Escherichia coli* (1)	6.4	
				*Klebsiella pneumoniae* (1)	6.4	
				*Proteus vulgaris* (1)	12.8	
				*Pseudomonas* spp. (1)	1.6	
				*Salmonella typhi* (1)	3.2	
				*Serratia marcescens* (1)	6.4	
				*Shigella sonnei* (1)	6.4	
			Fungi	*Aspergillus fumigatus* (1)	0.2	
				*Candida albicans* (1)	0.4	
				*Cryptococcus neoformans* (1)	0.4	
				*Trichophyton interdigitale* (1)	0.2	
				*Trichophyton mentagrophytes* (1)	0.2	
Chlorinated nitropyrroles	6	PM A	Gram-positive	*Bacillus anthracis* (1)	43	[25]
				*Staphylococcus aureus* (3)	43	
				*Staphylococcus epidermidis* (2)	85	
			Gram-negative	*Citrobacter freundii* (1)	85	
				*Escherichia coli* (4)	43–85	
				*Klebsiella pneumoniae* (2)	85	
				*Proteus* spp. (4)	43	
				*Pseudomonas aeruginosa* (1)	276	
				*Salmonella* spp. (4)	43	
				*Serratia marcescens* (1)	85	
				*Shigella sonnei* (1)	43	
			Fungi	*Aspergillus fumigatus* (1)	276	
				*Candida albicans* (1)	550	
				*Cryptococcus neoformans* (1)	138	
				*Trichophyton interdigitale* (1)	17.3	
				*Trichophyton mentagrophytes* (1)	69	
Brominated nitropyrroles	3	PM F	Gram-positive	*Bacillus subtilis* (1)	<0.4	[79]
				*Staphylococcus aureus* (1)	<0.7	
			Fungi	*Candida albicans* (2)	25	
				* Debaryomyces kloeckeri * (1)	43	
				* Helminthosporium sesamum * (1)	21	
				* Piricularia oryzae * (1)	<0.7	
				* Saccharomyces cerevisiae * (1)	43	
**Fluorinated derivatives**	**9**	**PM D**	**Gram-positive**	***Bacillus anthracis* (1)**	**0.1**	[80,81,82]
				***Staphylococcus aureus* (1)**	**≤0.4**	
				**MRSA (2)**	**0.2**	
			**Parasites**	***Toxoplasma gondii* (1)**	**IC_50_: 0.17**	[83]
			**Viruses**	**Zika virus**	**EC_50_** **: 6.0**	[84]
**Halogen-substituted derivatives**	**10**	**PM D**	**Gram-positive**	***Staphylococcus aureus* (4)**	<0.001 **IC_50_ biofilm: 0.003**	[37,44,60,64]
				*Staphylococcus epidermidis* (2)	<0.001	
				***Staphylococcus warneri* (1)**	**0.05**	
				***Staphylococcus xylosus* (1)**	**0.05**	
				***Pseudomonas aeruginosa* (2)**	**MBC: 30** **IC_50_ biofilm: 18.6**	
**Thiazolyl-halogenated derivatives**	**5**	**PM C**	**Gram-positive**	***Enterococcus faecalis* (1)**	**<0.2**	[62]
				**VRE (3)**	**<0.2**	
				**MRSA (2)**	**3.7**	
				***Streptococcus pyogenes* (1)**	**<0.2**	
			**Gram-negative**	***Acinetobacter baumannii* (1)**	**>472**	
				***Klebsiella pneumoniae* (1)**	**>472**	
				***Pseudomonas aeruginosa* (1)**	**>472**	
**Halogenated benzoyl–pyrazole derivatives**	**20**	**PM D**	**Gram-positive**	***Enterococcus faecalis* (1)**	**<0.4–1.5**	[82]
				***Staphylococcus aureus* (3)**	**0.2**	
				**MRSA (1)**	**0.2**	
				**VISA (1)**	**0.1**	

**Table 4:** Antimicrobial Activity of Natural Marinopyrroles. Findings since 2016 are bolded.

Notable Condition	Organism (# of Strains Tested)	Compound	Results/MIC/IC_50_ (μM)	Reference
Gram-positive	*Bacillus anthracis* (1)	MAR A	2–4	[87]
	***Bacillus subtilis* (3)**	**MAR A**	**0.2–3.7**	[61,87]
	***Enterococcus durans* (1)**	**MAR A**	**4**	[9]
	*Enterococcus faecium* VRE (2)	MAR A	0.1 (WHO-3)–250 (clinical isolate)	[54,87]
		MAR B	2.5–5	[87]
		MAR F	101	[87]
	***Enterococcus faecalis* (1)**	**MAR A**	**13.3**	[61]
	*Enterococcus faecalis* VRE (1)	MAR A	2	[54,87]
	***Enterococcus faecium* (1)**	**MAR A**	**8**	[61]
	***Listeria ivanovii* (1)**	**MAR A**	**0.5**	[61]
	*Staphylococcus aureus* (4)	MAR A	0.5 (NCTC8325) –2 (ATCC 29213)	[54,61,87]
	Hetero-GISA	MAR A	0.5–1	[87]
	MRSA (10)	MAR A	0.3 (unknown)–**1 (2 strains)**	[7,40,54,61,87]
		MAR B	0.3 (3 strains CA-MRSA, 1 strain HA-MRSA)–1 (unknown)	[40,87]
		MAR C	0.3	[40]
		MAR F	3.2 (3 strains CA-MRSA) 6.5 (unknown) >96 (1 strain HA-MRSA)	[40,87]
	ORSA (1)	MAR A	2.0	[54]
	VISA (2)	MAR A	0.5–1	[87]
	VRSA (2)	MAR A	0.7–3	[87]
	*Staphylococcus epidermidis* (3)	MAR A	<0.4 (2 strains)–5.3 (ATCC 14990)	[9,61,87]
	MRSE (1)	MAR A	0.1–2	[54]
	*Streptococcus* *agalactiae* (1)	MAR A	4	[87]
	***Streptococcus*** ***pneumoniae* (1)**	**MAR A**	**0.3**	[61]
	*Streptococcus pyogenes* (2)	MAR A	2	[87]
Gram-negative	***Acinetobacter baumannii* (1)**	**MAR A**	**>64**	[61]
	***Campylobacter jejuni* (1)**	**MAR A**	**4**	[9]
	*Escherichia coli* (3)	**MAR A**	**>64**	[9,54,61,87]
	Resistant *Escherichia coli* (4)	MAR A	>250	[88]
	*Haemophilus influenzae* (2)	**MAR A**	**0.1 (ATCC 49247)–4 (ATCC 1021)**	[9,87]
	***Klebsiella aerogenes* (1)**	**MAR A**	**>64**	[61]
	Resistant *Klebsiella pneumoniae* (2)	**MAR A**	**>128 (ESBL + clinical isolates)** >16 (ATCC 700603)	[54,61,87,88]
	***Moraxella catarrhalis* (1)**	**MAR A**	**1.2**	[9]
	***Mycobacterium smegmatis* (1)**	**MAR A**	**50–100**	[89]
	***Mycobacterium tuberculosis* (1)**	**MAR A**	**25**	[89]
	***Neisseria gonorrhoeae* (1)**	**MAR A**	**<0.4**	[9]
	***Neisseria meningitidis* (1)**	**MAR A**	**4**	[9]
	***Neisseria mucosa* (1)**	**MAR A**	**11.8**	[9]
	***Ochrobactrum antrhopi* (1)**	**MAR A**	**32**	[61]
	***Proteus mirabilis* (1)**	**MAR A**	**>98**	[9]
	***Providencia alcalifaciens* (1)**	**MAR A**	**>64**	[61]
	*Pseudomonas aeruginosa* (2)	**MAR A**	**>64**	[54,61,87]
	***Salmonella enterica* (1)**	**MAR A**	**>64**	[61]
	***Shigella sonnei* (1)**	**MAR A**	**>64**	[61]
	***Vibrio cholerae* (1)**	**MAR A**	**>64**	[61]
	***Yersinia pseudotuberculosis* (1)**	**MAR A**	**>64**	[61]
Parasites	***Toxoplasma gondii* (1)**	**MAR A**	**IC_50_: 0.3**	[83]

**Table 5:** Antimicrobial Activity of Marinopyrrole Derivatives. Findings since 2016 are bolded.

Compounds/Series Tested	# of Analogs Tested	Derivative Source	Classification	Organism	Best MIC/IC_50_ (μM)	Reference
Symmetrical derivatives	17	MAR A	Gram-positive	*Staphylococcus aureus* (1)	12.9	[54,56,87,88,97]
				MRSA (4)	0.6	
				*Staphylococcus epidermidis* (1)	3.7–15	
				MRSE (1)	1.1–8	
				ORSA (1)	6.5	
				VRE (3)	0.8	
			Gram-negative	*Escherichia coli* (1)	<207	
				*Klebsiella pneumoniae* (1)	<207	
				*Pseudomonas aeruginosa* (1)	<207	
10,10′-bis(trifluoromethyl) derivatives	23	MAR A	Gram-positive	*Staphylococcus aureus* (1)	0.2	[49,54,97]
				**MRSA (2)**	**0.2–0.4**	
				MRSE (1)	<0.01	
				ORSA (1)	1.5	
				VRE (1)	0.8	
			**Viruses**	**Coxsackievirus (2)**	**IC_50_: 0.01**	[48,96]
				**Enterovirus (2)**	**IC_50_: 0.02**	
				**Dengue Virus (1)**	**IC_50_: 0.15**	
				**Japanese Encephalitis Virus (1)**	**IC_50_: 0.05**	
				**Yellow Fever Virus (1)**	**IC_50_: 0.05**	
				**Zika Virus (1)**	**IC_50_: 0.07**	
Asymmetrical derivatives	3	MAR A	Gram-positive	MRSA (2)	2.5–5	[56,87]
				VRE (1)	5.8–11.6	
Asymmetrical monochlorinated derivatives	4	MAR A	Gram-positive	MRSA (1)	0.4	[53,83]
			**Parasites**	***Toxoplasma gondii* (1)**	**IC_50_: 0.16**	
**Pyrrolomycin-like derivatives of MAR A**	**2**	**MAR A & PM D**	**Gram-positive**	**MRSA (1)**	**0.2**	[80,83]
			**Parasites**	***Toxoplasma gondii* (2)**	**IC_50_: 0.09**	

## Data Availability

The original contributions presented in the study are included in the article. Further inquiries can be directed to the corresponding author.
